# Diastolic dysfunction evaluation by cardiovascular magnetic resonance derived E, a, e’: Comparison to echocardiography

**DOI:** 10.14814/phy2.70078

**Published:** 2024-11-27

**Authors:** Jérôme Lamy, Jie Xiang, Nimish Shah, Jennifer M. Kwan, Yekaterina Kim, Krishna Upadhyaya, Samuel W. Reinhardt, Judith Meadows, Robert L. McNamara, Lauren A. Baldassarre, Dana C. Peters

**Affiliations:** ^1^ Department of Radiology and Biomedical Imaging Yale University New Haven Connecticut USA; ^2^ PARCC INSERM, Université Paris Cité | Hôpital Européen Georges Pompidou, APHP Paris France; ^3^ Department Biomedical Engineering Yale University New Haven Connecticut USA; ^4^ Cardiovascular Division, Department of Internal Medicine Yale University New Haven Connecticut USA

**Keywords:** diastolic dysfunction, echocardiography, MRI, phase‐contrast

## Abstract

Transthoracic echocardiography (TTE) is the first‐line and most useful imaging modality for evaluating diastolic dysfunction (DD). Cardiovascular magnetic resonance (CMR) has not been fully evaluated for this task. We investigated the utility of CMR for DD evaluation.Thirty‐one patients with a recent TTE (within 4 months) were prospectively enrolled, along with 12 healthy age‐matched subjects. CMR imaging was performed at 1.5 T to assess diastolic function by quantifying mitral inflow velocities (E and A), mitral annular velocities (e’), and left atrial volume (LAVi). Measurements by TTE and CMR were compared using regression. The diagnostic accuracy of CMR for DD was determined.CMR derived E, A, E/A, e’ and E/e’ all correlated moderately to strongly with TTE, and more strongly when comparing studies performed closer in time (E: *r* = 0.68, E deceleration time: *r* = 0.82, A: *r* = 0.78, e’ *r* = 0.75, E/e’: *r* = 0.80, *p* = 0.001; LAVi: *r* = 0.79, *p* < 0.001; E/A: *r* = 0.82, *p* < 0.001, *n* = 14 within 45 days). Using CMR criteria analogous to TTE, there was 82% (23/28) agreement regarding the presence of DD (95% CI [63 to 93%]), with 100% sensitivity and 75% specificity, and 71% (20/28) agreement in the absolute DD grade.CMR can evaluate diastolic function, with overall strong agreement to TTE.

## 1 INTRODUCTION

Diastolic dysfunction (DD) reflects impaired filling of the left ventricle (LV) in diastole due to myocardial stiffness, causing reduced capability to quickly relax its end‐diastolic position (Mitter et al., 2017). The reduced left ventricular (LV) compliance leads to increased LV filling pressures and ultimately heart‐failure with preserved ejection fraction (HFpEF), an increasingly common form of HF with no specific treatment (Chung et al., 2015) (Chetrit et al., 2020; Savarese et al., 2023). Therefore, assessment of DD holds crucial importance as a valuable prognostic tool, for improving the patient care. Early detection of DD is paramount given the fact that early stages are asymptomatic and there is a risk of progression to HF.

Despite the importance of DD assessment, cardiovascular magnetic resonance (CMR) tools for evaluation of DD are not fully developed. Transthoracic echocardiography (TTE), with its widespread availability and operational simplicity, stands as the first‐line non‐invasive imaging technique for DD evaluation. CMR approaches (Ibrahim et al., 2021; Rajiah et al., 2023) include tagging, phase‐contrast (PC) (including 4D Flow), and volumetric analyses of LV and left atrial (LA) cine images to obtain filling rates (Backhaus et al., 2021; Kawaji et al., 2009; Mendoza et al., 2010) or LA strains (Backhaus & Schuster, 2018; Nguyen et al., 2021). Additionally, other studies have used CMR to measure metrics equivalent to those by TTE, including studies of E and A by 2D PC (Calkoen et al., 2015; Hartiala et al., 1993; Rathi et al., 2008) and 4D Flow (Brandts et al., 2011).

There are extremely limited studies of mitral inflow and valve velocity parameters E, A, and e’ by CMR for direct comparison with TTE (Fujikura et al., 2024; Ramos et al., 2020; Seemann et al., 2018); the earliest study was retrospective. Only two recent studies (Fujikura et al., 2024; Ramos et al., 2020) have attempted to diagnose DD grade. In summary, CMR's capability to evaluate LVDD is still being validated, and data is rare.

The purpose of this prospective study is to compare LVDD metrics by CMR (E, A, e’, and LAVI, and deceleration times, E DT) versus TTE, and compare LVDD diagnostic grade using CMR versus TTE. The techniques used include measurement of mitral velocity, e’, using deep learning tracking (Gonzales et al., 2021) from long‐axis cine, and 2D PC measurements of E and A waves. A CMR assessment of LVDD was developed and evaluated. We hypothesized a strong relationship between equivalent metrics of diastolic function (including E, A, and e’, etc.) and good agreement regarding diastolic grade between CMR and TTE.

## 2 MATERIALS AND METHODS

### 2.1 Subjects

The study was approved by our local IRB, the Yale Human Investigation Committee, and all subjects provided written and informed consent. Adults (>18 years old) with TTE which evaluated DD within 4 months (average 44 ± 41 days) of a scheduled CMR were eligible. Exclusion criteria were LVEF <50% by TTE, arrhythmia during the scan or a major event between TTE and CMR. Patients with HFrEF were excluded since they according to the guidelines have LVDD; their inclusion would artificially improve MRI's detection accuracy. Thirty‐one patients were identified by clinicians and were prospectively and consecutively enrolled from October 2019 to August 2022. All subjects underwent a clinical CMR exam at 1.5 T Siemens scanner (Aera, Erlangen, Germany).

Patients with findings of normal LVDD nevertheless had a clinical indication for CMR, so their LVDD measurements might not reflect a healthy cohort. In order to have a cohort of healthy subjects for determining normal reference values, we prospectively enrolled 12 age‐matched healthy controls who were imaged with the same protocol on a 3 T Siemens scanner. In addition, also at 3 T, 10 healthy younger subjects were enrolled in an abbreviated protocol to measure scan‐rescan reproducibility of E, A, and e’. PC performs with good reproducibility between these two field strengths (Lotz et al., 2005), as does cine, and we do not expect that field strength can impact quantitation of diastolic metrics.

### 2.2 CMR imaging

**FIGURE 1:**
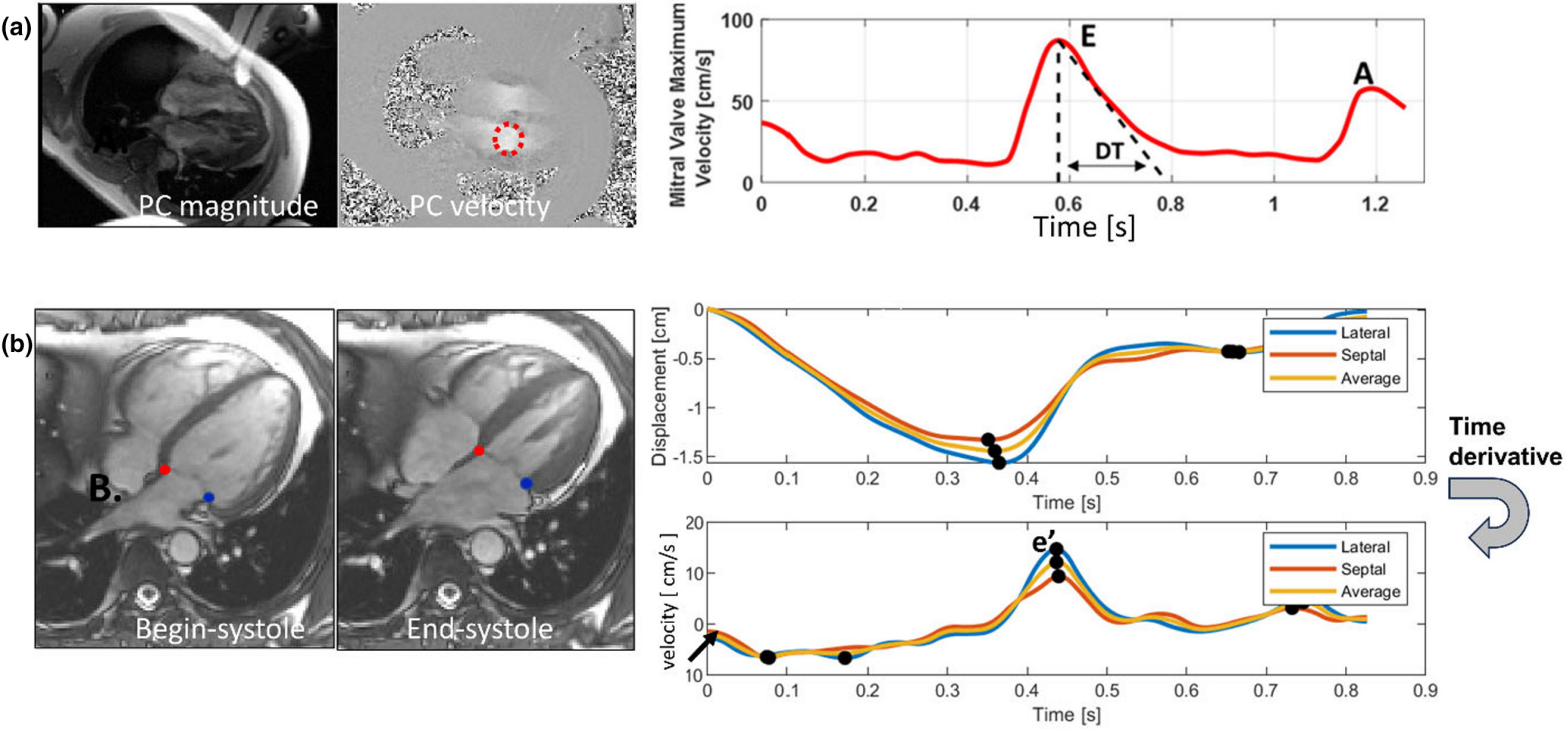
(a) PC images of the 4 chamber LV for the mitral valve maximum velocities (E and A and deceleration time DT) in the red circular ROI. (b) MVnet^4^ automatically analyzed cines throughout the cardiac cycle, identifying mitral valve insertion points (blue circle = lateral insertion point; red circle = septal insertion point) from 4 chamber cines. These positions were post processed to measure phasic mitral valve insertion point excursion and e’, mitral valve tissue velocity during early diastole (both septal and lateral).

Patients and healthy controls underwent a standard CMR exam (including long‐axis 2ch and 4ch cines) supplemented with a 4 chamber (4ch) in‐plane PC with flow‐encoding in the LV long‐axis direction (Figure 1). Altogether, additional scan time required was 1 breath‐hold (and <5 min for all needed scans, long‐axis cines, and PC). For the long‐axis cines, the scan parameters were: Breath‐hold, retrospectively ECG‐gated bSSFP, TR/TE/θ = 2.4 ms/1.2 ms/50°–60°, 36 ms temporal resolution, 8 mm slice, 208×168 matrix, 320×320mm FOV (1.5×1.9 mm^2^in‐plane resolution). The long‐axis 4ch PC sequence used in‐plane velocity encoding, parallel to the long‐axis with a VENC of 150 cm/s. Scan parameters were: 2D GRE breath‐hold, retrospective ECG‐gating, TR/TE/θ = 5.9 ms/2.5 ms/20°, temporal resolution of 36 ms, 192×115 matrix, 380×278 mm FOV (2.0×2.4 mm^2^ in‐plane resolution), 6–8 mm slice thickness. The long‐axis PC approach permits measurement of E and A which is less sensitive to valve‐motion, unlike the short axis PC method (Brandts et al., 2011; Paelinck et al., 2004) for E and A.

### 2.3 TTE

TTE was performed using the standard clinical protocol for a complete exam including spectral Doppler and color Doppler with Philips iE33 X5 1–5 MHz and Epiq machines (Philips, Amsterdam, Netherlands). Views were obtained from the parasternal long axis and short axis, as well as the apical 4‐chamber, 2‐chamber, 3‐chamber, subcostal, and suprasternal notch. For diastolic function, mitral inflow pulse‐wave Doppler was obtained in the apical 4‐chamber view at the level of the leaflet tips with E and A wave velocities measured by experienced sonographers. The medial and lateral mitral annular tissue Doppler velocities (e’) were obtained from the apical 4‐chamber view and measured by the sonographer. Doppler measurements were averaged over several heart beats. LA volume was calculated using the biplane method in the apical 4‐chamber and apical 2‐chamber views. Tricuspid regurgitant jet peak velocity was measured by the sonographer in the parasternal RV inflow, parasternal short axis, and apical 4‐chamber views (occasionally also the reverse apical 4‐chamber view), with the maximum velocity included in the final report and used for diastolic function assessment.

### 2.4 Image analysis

CMR LV functional parameters were obtained from patients' MRI reports. Image analysis of all PC sequences and cine images was performed, blinded to TTE results, using the freely available software Segment v3.2 R8836 (Medviso, segment.heiberg.se) (Heiberg et al., 2010). Analyses were performed by a reader with >5 years of CMR experience. Mitral valve peak flow maximal velocities E and A, in early and late diastole respectively were evaluated from the PC images by drawing a circular area (radius 2 cm) at the level of the valve (Figure 1a), with E and A obtained as the peak velocity within the ROI. Deceleration time of the E wave was measured, analogously to TTE, as the time interval from the peak velocity to zero velocity, extrapolating the initial downward slope of the E wave to zero (Figure 1a). Lateral and septal e’ were automatically evaluated by a deep learning framework (Mvnet, previously trained in 700 subjects) enabled as a plug‐in to Segment (Gonzales et al., 2021) on standard 4ch cine images (Figure 1b). Both e’ values were also averaged to calculate the E/e'_ave_ ratio. LA volume was measured using area‐length biplane method (Wylie Jr. et al., 2008): LA volume = 0.85·(LA Area_2CH_ ·LA area_4CH_)/max(LA diameter), and was indexed to BSA (LAVi). All metrics were compared to their TTE counterparts. We further categorized TTE studies acquired closer in time to the CMR (<45 days vs. ≥45 days), to determine how this impacted CMR versus TTE correlations.

### 2.5 Diastolic dysfunction grading

TTE DD grading was determined for each patient, according to the 2016 ASE/EACVI criteria (Nagueh et al., 2016), and super‐categorized as presence or absence of DD, with indeterminate cases categorized as DD. TTE grading was adjudicated by a Level III trained echocardiographer. Specific CMR cutoffs that best represented each TTE cutoff used for diagnosing LVDD (Nagueh et al., 2016) were determined using ROC analysis for analogous grading of DD by CMR for the following parameters: E, A, e’ septal and lateral, E/e’, and E/A. Diastolic grading by CMR incorporated TTE measurement of TR velocity, since it was not measured by CMR.

### 2.6 Multiple logistic regression

We also performed multiple logistic regression. We added all clinical and CMR variables that had a univariate *p*‐value to predict presence of LVDD by TTE of *p* < 0.5, and used backward selection to generate a model. We performed leave‐one‐out analysis to determine the accuracy of the model.

### 2.7 Scan‐rescan reproducibility

Ten (seven for e’) healthy subjects (50% female, age 36 ± 15 years) were scanned and rescanned using the long‐axis PC‐GRE and long‐axis 4‐chamber bSSFP cine (within the same session, without repositioning the slice), to remeasure E, A, and e’. Regression and Bland–Altman analysis were performed to evaluate reproducibility.

### 2.8 Statistics

Statistical analyses were performed using JMP‐SAS software 16.2 (Cary, North Carolina). All continuous variables are presented as mean ± standard deviation. Data was tested for normality using a quantile plot (E, E DT, A, E/A, e’, and E/e’), and was found normally distributed. Metrics of DD were compared using Pearson correlation, linear regression and Bland–Altman analyses were performed (mean ± 1.96*standard deviation of the differences). For Pearson correlations, 0.2–0.39 was considered weak, 0.40–0.59 as moderate, 0.6–0.79 as strong, and 0.8–1 as very strong. ROC analysis was used to determine cutoff values and AUC for CMR variables (e’ septal and lateral, maximum LAVi, E/e’ and E/A) from diagnostic TTE thresholds. Comparison of DD categories was performed using Cohen's kappa statistic. Logistic regression with leave‐on‐out analysis was performed in scikit‐learn (1.4.1). The sample size (*N* = 30) was planned to demonstrate a correlation between TTE and CMR for E/e’ of 0.8 with 95% CI [0.6–0.9].

## 3 RESULTS

### 3.1 Population

Among the 31 subjects 11 patients underwent CMR for ectopy, arrythmia or a conduction abnormality. Twelve were imaged to identify a cardiomyopathy such as hypertrophic cardiomyopathy. One subject underwent CMR due to LV hypertrophy, 1 due to a suspected cardiac mass, 2 for infiltrative disease, 3 for myocarditis, and 1 for unidentified reasons. Three patients had missing CMR or TTE data, limiting the DD grading to 28 patients.

### 3.2 Scan‐rescan reproducibility of E, a, and e’ by CMR

Reproducibility was strong in healthy subjects using scan‐rescan imaging (during the same exam), with Bland–Altman limits of agreement of 1.2 ± 13.2 cm/s for E, 0.0 ± 9.8 cm/s for A, and 0.5 ± 2.1 cm/s for e’. The correlation coefficients between the repeated measurements were *r* = 0.93 for E (ICC = 0.92) and *r* = 0.94 for A (ICC = 0.94), and *r* = 0.93 for e’ (ICC = 0.92).

### 3.3 Comparison between TTE and CMR parameters

Table 1 describes basic demographics of the volunteers and patients. Patients with DD were older. LVEF by TTE and CMR correlated only moderately (*r* = 0.47, *p* = 0.01). Table 2 presents the TTE and CMR variables E, E/A, e’, E/e’ for the 31 patients with and without LV DD by TTE, and the CMR values from our healthy cohort. Among the 28 patients with complete TTE and CMR studies, 8 had abnormal TTE findings (*n* = 1 grade 1, *n* = 2 grade 2, *n* = 1 grade 3, *n* = 4 indeterminate). Table 2 indicates which parameters by both CMR and TTE were significantly different between patients with and without TTE‐defined DD. Notably, TTE and CMR‐derived e’ values were lower in DD and LAVi by CMR was larger in DD (but not by TTE). Both TTE and CMR E/e’ values were higher in patients with DD.

**TABLE 1:** Basic characteristics.

	Controls (*n* = 12)	Patients no LVDD by TTE (*n* = 22)	Patients LVDD by TTE (*n* = 9)
Sex [M/F]	5/7	8/13	2/7
Age [year]	46 ± 17	49 ± 16**	63 ± 9
BMI [kg/m^2^]	26.8 ± 5.7	26.9 ± 5.8	28.4 ± 3.9
LV EF [%]		58 ± 4	59 ± 5
LVEDVi [mL/m^2^]		86 ± 14	85 ± 30
LVESVi [mL/m^2^]		50 ± 8	54 ± 14
LVMi [g/m^2^]		52 ± 17	57 ± 13
Blood pressure systolic/Diastolic (mmHg)		126 ± 21/73 ± 9	140 ± 19/ 77 ± 5

^**^ Age significantly different, *p* = 0.003.

**TABLE 2:** CMR and TTE diastolic indices.

Mitral flow velocities	Controls (*n* = 12)	Patients no LVDD by TTE (*n* = 22)		Patients LVDD by TTE (*n* = 9)		CMR versus TTE (all subjects)	
	CMR	TTE	CMR	TTE	CMR	Bias ± 1.96SDs	*R* (*p*)
E [cm/s]	79 ± 9	76 ± 17	73 ± 13	80 ± 28	70 ± 15	−4.9 ± 25	0.62 (<0.001)
A [cm/s]	62 ± 15	62 ± 18	61 ± 16	72 ± 18	68 ± 18	−2.6 ± 31	0.76 (<0.001)
E DT [ms]	234 ± 65	200 ± 66	225 ± 70	230 ± 89	255 ± 71	−35 ± 118	0.58 (0.002)
E/A	1.37 ± 0.46	1.26 ± 0.36	1.29 ± 0.39	1.19 ± 0.45	1.09 ± 0.32	−0.02 ± 0.55	0.70 (<0.001)
e’ septal [cm/s]	9.3 ± 2.9	9.9 ± 3.3	7.6 ± 2.6	6.1 ± 1.9**	4.7 ± 1.9*	−2.2 ± 4.9**	0.69 (<0.001)
e’ Lateral [cm/s]	10.9 ± 3.8	13.0 ± 4.4	9.6 ± 2.8	8.3 ± 2.5**	6.4 ± 1.7**	−3.1 ± 7.5**	0.54 (0.002)
e’ Average [cm/s]	10.1 ± 3.2	11.5 ± 3.6	8.6 ± 2.4	7.2 ± 2.0**	5.6 ± 1.6**	−2.7 ± 5.3**	0.7 (<0.001)
E/e’	8.6 ± 3.5	7.7 ± 2.9	9.4 ± 3.6	12.1 ± 4.3*	12.8 ± 3.8*	1.8 ± 6*	0.67 (<0.001)
Other							
LAVi[ml/m^2^]	—	29 ± 10	33 ± 13	36 ± 12	54 ± 19*	12 ± 27	0.54 (0.007)
TR velocity [cm/s]	—	208 ± 28		272 ± 26*			

*Note*: Significant differences between measurements in patients with and without diastolic dysfunction (as determined by TTE) indicated by * (*p* < 0.05) and **(*p* < 0.001). For comparing CMR versus TTE, Pearson correlations and Bland–Altman differences (CMR‐ECHO) ± 1.96SD are shown, with significant differences between CMR and TTE indicated by *(*p* ≤ 0.05) and **(*p* < 0.001).

Abbreviations: BMI, body mass index; CMR, cardiac magnetic resonance; DT, deceleration time; LAVi, left atrial maximum volume indexed over body surface area; LVDD, left ventricular diastolic dysfunction; LVEF, left ventricular ejection fraction; TR, tricuspid regurgitation; TTE, transthoracic echocardiography.

**FIGURE 2:**
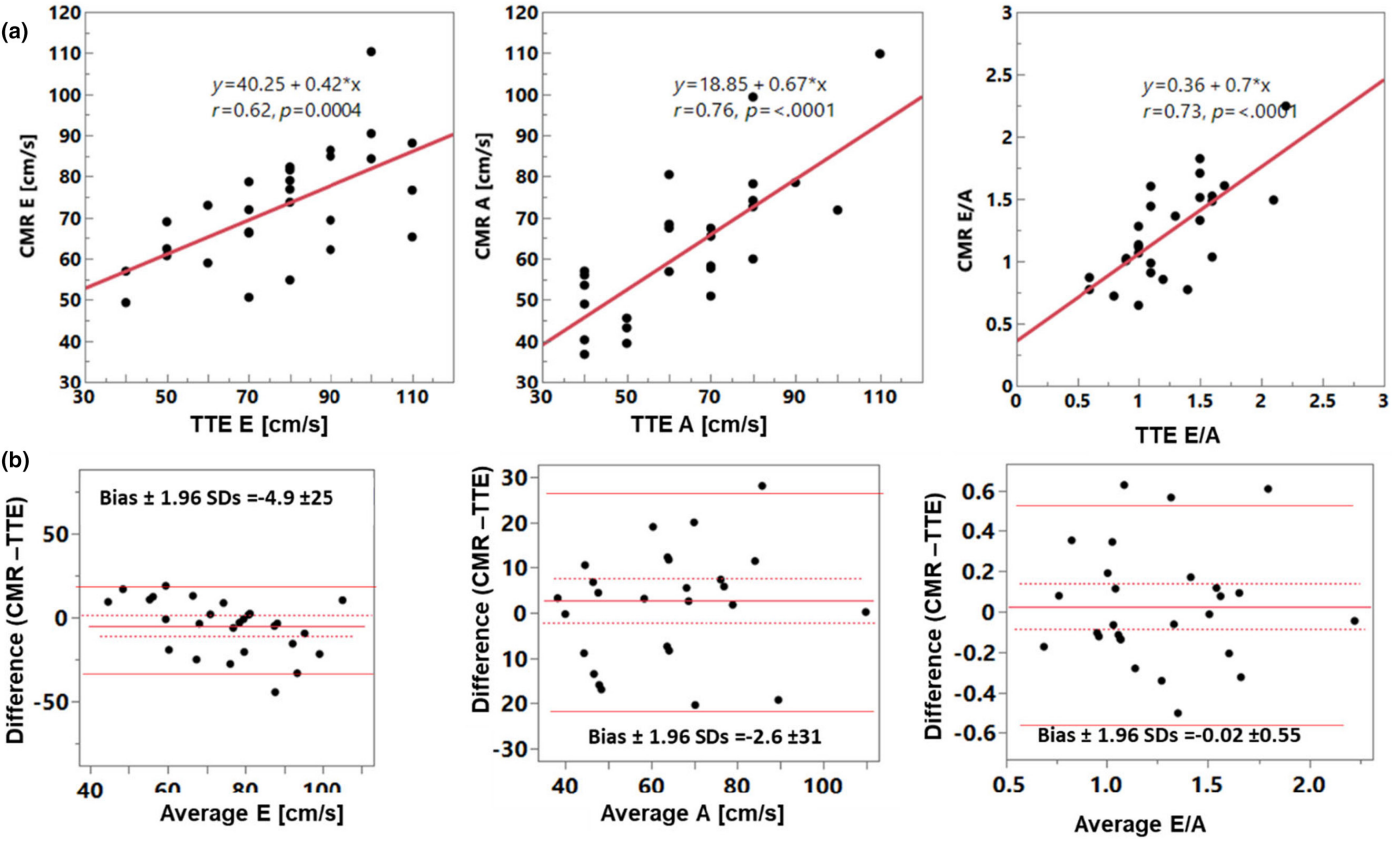
Comparison between TTE and CMR data showing regression (a) and Bland–Altman (b) plots, including E, A and E/A. TTE, transthoracic echocardiography. CMR, cardiovascular magnetic resonance. Solid lines show Bland–Altman limits of agreement.

**FIGURE 3:**
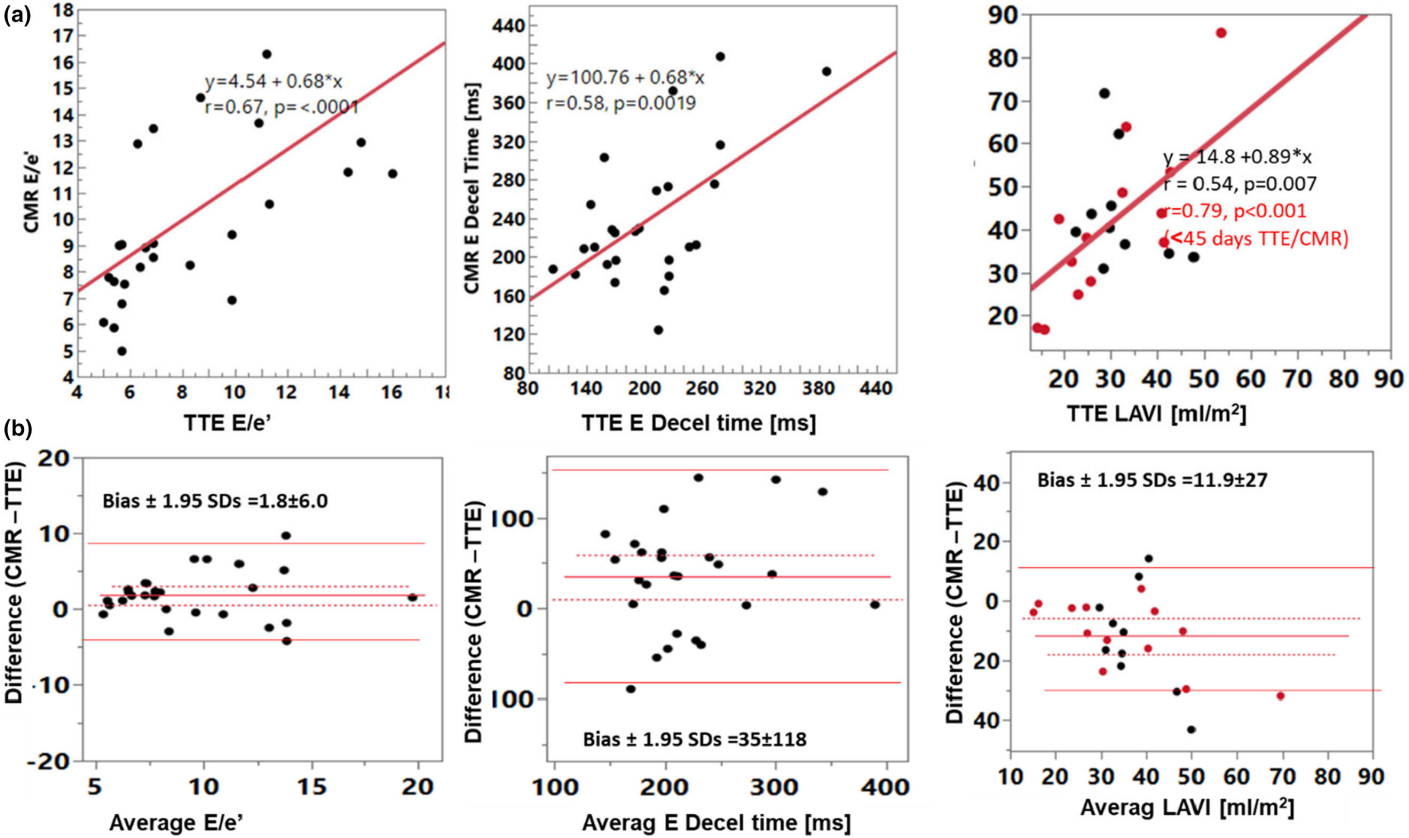
Comparison between TTE and CMR data showing regression (a) and Bland–Altman (b) plots, including E/e’, E wave deceleration time (DT), and LAVi. Red markers show LAVi data for subjects with TTE within 45 days of CMR, where including only studies close in time improved the relationship for LAVi. LAVi, left atrial volume indexed by BSA. Decel, deceleration. Solid lines show Bland–Altman limits of agreement.

**FIGURE 4:**
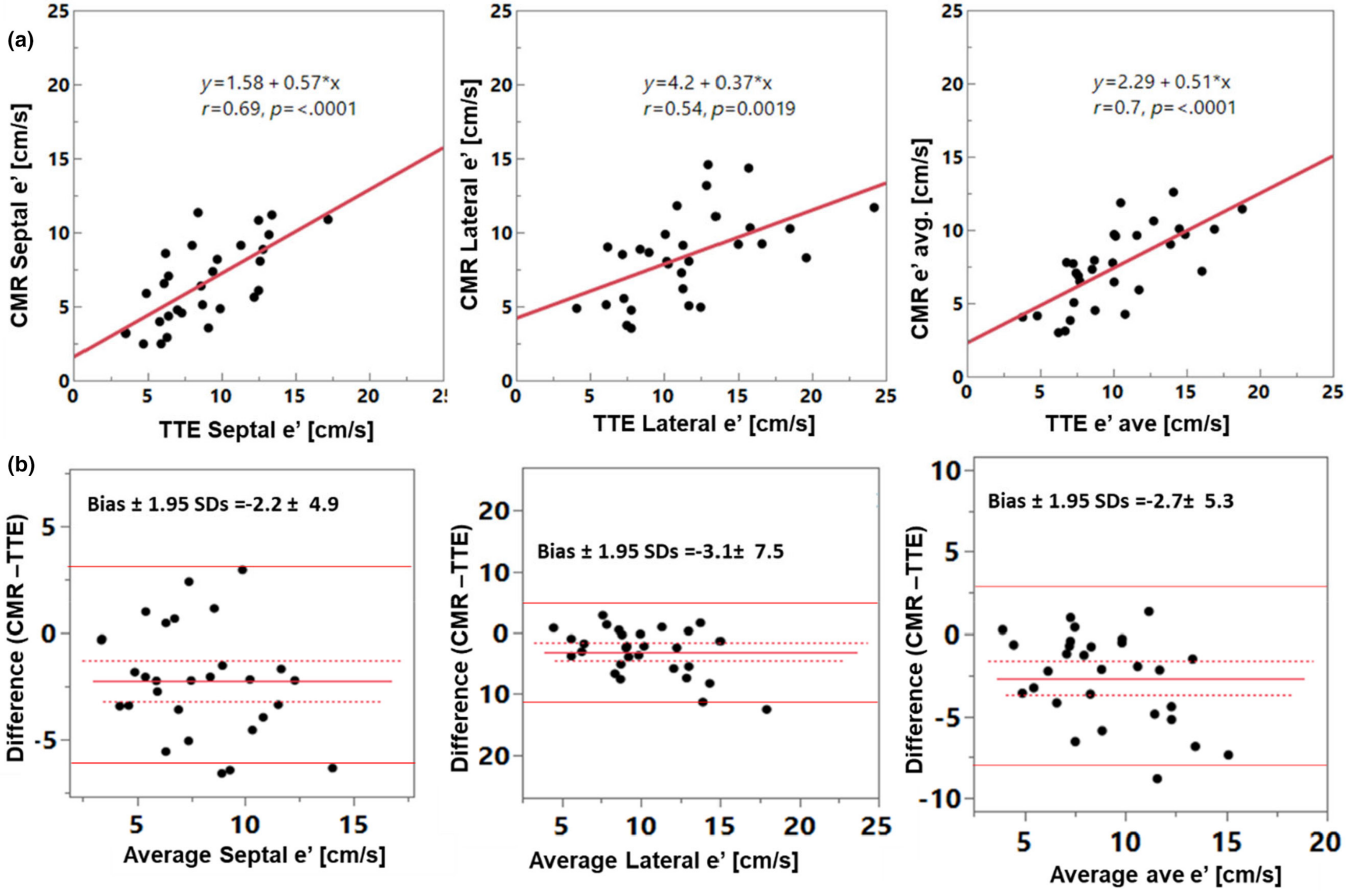
Comparison between TTE and CMR data showing regression (a) and Bland–Altman (b) plots, including e’ septal, e’ lateral, and e’ average. Solid lines show Bland–Altman limits of agreement.

**FIGURE 5:**
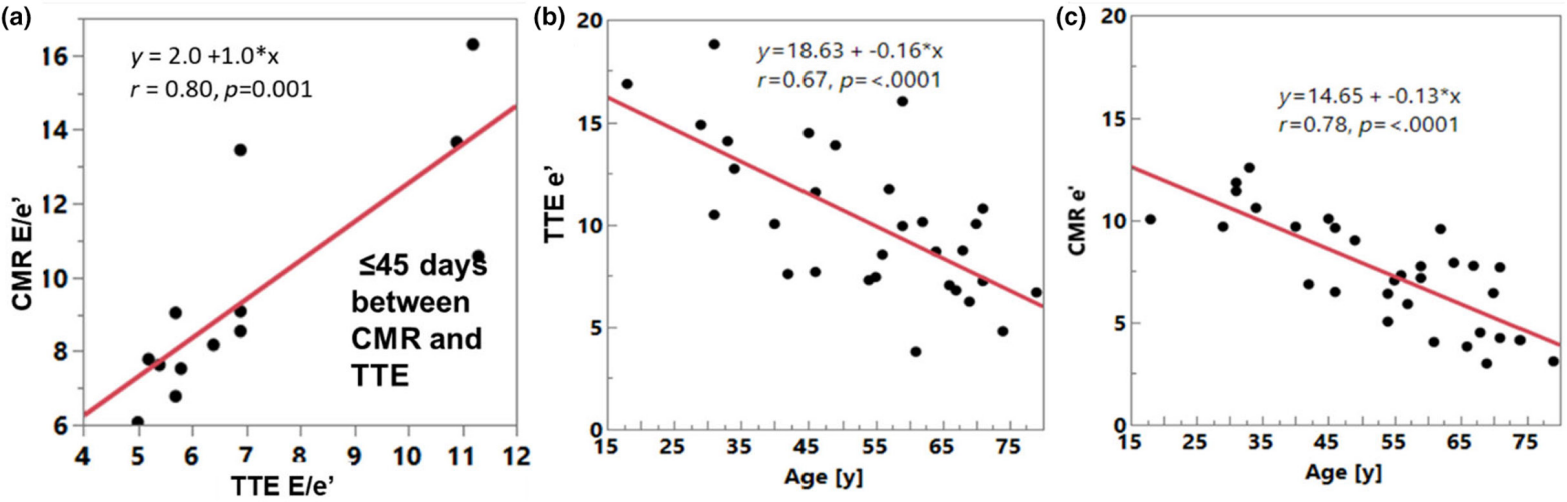
Comparison between TTE and CMR data, including (a) E/e’ correlation, for studies that were acquired close in time. (b,c) e’ showed expected relationship with age, but this was stronger by CMR compared to TTE.

There were moderate to strong correlations between TTE and CMR (*r* > 0.6, *p* < 0.002) for E, A, E/A, e’, E/e’ (Table 2 and Figures 2, 3, 4). LAVi showed weaker correlation (*r* = 0.54, *p* = 0.007). Notably, analyzing only patients who underwent TTE and CMR studies with a shorter time gap, within 45 days (*n* = 14), some key associations became stronger, with the following *r*‐values: E: 0.68, E decel time: *r* = 0.82, A: *r* = 0.78, E/A: *r* = 0.75, e’: *r* = 0.75, E/e’: *r* = 0.8 (Figure 5a), LAVi: *r* = 0.79 (Figure 3, red markers); lateral e’: *r* = 0.55, septal e’: *r* = 0.69 (all *p* < 0.05). The direct comparison of paired values by CMR and TTE found low and insignificant biases for E, and A, but CMR's e’ velocities were significantly lower compared to TTE (bias −2.7) (Table 2). Additionally, LA volumes were larger by CMR (bias 11.9 mL/m^2^).

**FIGURE 6:**
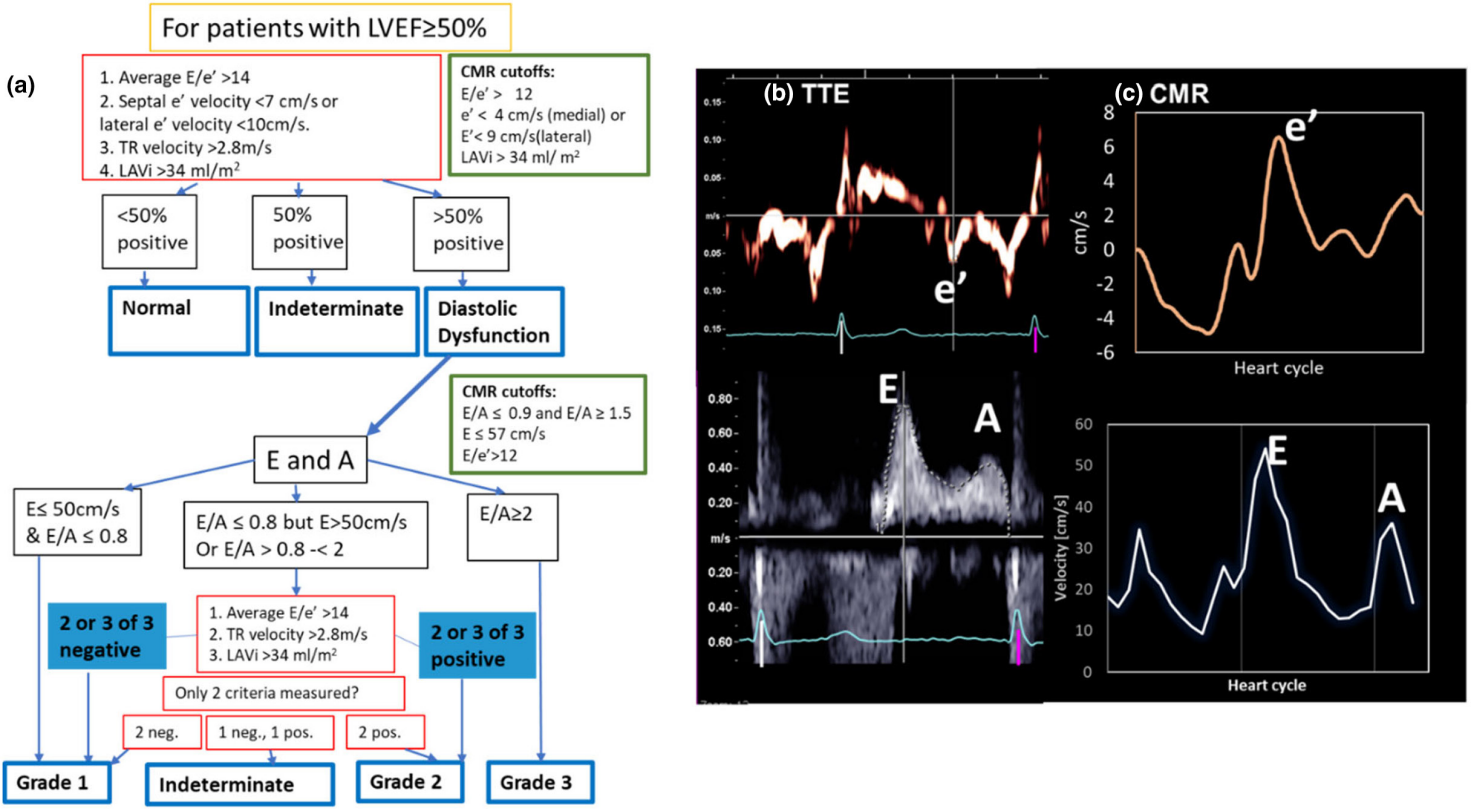
(a) TTE diastolic grading criteria, with CMR‐based cutoffs shown for this patient group (as determined in Table 3). (b) TTE and (c) CMR data in a patient with grade 3 diastolic dysfunction, by both modalities. The mitral valve velocity curves showing e’ (top) and mitral inflow curves showing E and A waves are displayed (bottom). By TTE, *E* = 80 cm/s, *A* = 40 cm/s, e’ = 9 cm/s, E/e’ = 10, E/A = 2 and TR = 300 cm/s. By CMR, *E* = 55 cm/s, *A* = 37 cm/s, e’ = 8 cm/s, E/e’ = 8.2, E/A = 1.5.

### 3.4 Age and e’

e’ decreased with age with a stronger association observed for CMR‐derived e’ in comparison to TTE (e’ average vs. age: TTE: *r* = −0.67, *p* < 0.001 and CMR: *r* = −0.78, *p* < 0.001) (Figure 3c).

### 3.5 Cutoffs for CMR grading of diastolic dysfunction

TTE uses thresholds in its criterion for the evaluation of the DD (Figure 6a). CMR cutoffs specific to our cohort were independently determined for each parameter using a ROC analysis. This analysis provided a CMR parameter cutoff best predicting the corresponding TTE criterion. CMR parameter cutoffs in comparison to TTE cutoffs are displayed in Table 3, along with AUC values, sensitivity and specificity, and the CMR values corresponding to TTE cutoffs based on simple the linear fits (e.g., using the linear relationship between CMR and TTE to identify equivalent CMR cutoffs).

**TABLE 3:** CMR cutoffs corresponding to TTE values, used in evaluation of LVDD according to current guidelines (Nagueh et al., 2016).

TTE cutoff	CMR cutoff^a^	AUC	Specificity/Sensitivity	CMR analog to TTE^b^
e’ septal<7	e’ septal≤4	0.81	95%, 50%	6.6 cm/s
e’ lateral<10	e’ lateral≤9	0.80	60%,100%	7.9 cm/s
E ≤ 50 cm/s	E ≤ 57 cm/s	0.96	92%,100%	61 cm/s
E/e’ > 14	E/e’ > or = 12	0.83	74%,100%	14
E/A > 2	E/A ≥ 1.5	0.89	77%,100%	1.8
E/A < 0.8	E/A ≤ 0.9	0.92	88%, 100%	0.91
LAVi>34 mL/m^2^	LAVI≥34 mL/m^2^	0.61	36%, 100%	45.1 mL/m^2^

^a^ CMR analogs to TTE cutoff values based on AUC analysis (and used in Table 4).

^b^ CMR analogs to TTE cutoff values, based on linear fits.

### 3.6 Diastolic dysfunction grading by CMR

Figure 6a shows the DD criteria by TTE superimposed with the criteria by CMR obtained for this cohort (based on ROC analysis), and using the current 2016 ASE/EACVI guidelines (Nagueh et al., 2016). CMR DD grading used TTE TR jet velocity values. Table 4 is a contingency table showing the extent of agreement between TTE and CMR in diagnosis of DD. In 82% (23/28) (95% CI [63%–93%]) of patients, there was agreement between CMR and TTE regarding the presence of any DD, with sensitivity 100% (95% CI [63%–100.0%], specificity 75% (95% CI [51%–91%] (with “indeterminate” DD considered as DD). Cohen's kappa of 0.63 indicated moderate agreement. There was a 71% (20/28) absolute agreement in the DD grade (*κ* = 0.49, *p* < 0.0001). CMR detected more dysfunction (13 vs. 8 non‐normal cases) and mismatched grades were systematically higher using CMR‐derived criteria. The time between CMR and TTE exams did not explain the difference in diastolic gradings: the average time difference was 48 ± 37 days in studies which agreed fully, and 50 ± 34 days in studies which disagreed.

**TABLE 4:** Contingency table showing CMR versus TTE agreement regarding diastolic dysfunction grading, showing 82% CMR accuracy for any DD (including indeterminate) and 71% accuracy regarding absolute DD grade. Indet, indeterminate.

CMR TTE	Normal	Indet.	Grade 1	Grade 2	Grade 3
Normal	15	4	1	0	0
Indeterminate	0	1	1	1	1
Grade 1	0	0	1	0	0
Grade 2	0	0	0	2	0
Grade 3	0	0	0	0	1

The most conspicuous disagreement was observed in a patient with suspected cardiotoxicity induced myocarditis. Both TTE and CMR were imaged the same day and while TTE reported indeterminate DD (high TR jet velocity and enlarged LAVi), CMR graded the patient as grade 3 DD. Another patient presented with heart‐block and a possible diagnosis of sarcoidosis, initially indeterminate by TTE was later assessed as grade 2 by CMR. Another was normal by TTE, Grade 1 by CMR, and had hypertrophic cardiomyopathy which required ICD implantation a month later. Figure 6b shows an example where both CMR and TTE identified grade 3 DD.

### 3.7 Multiple logistic regression for the presence of LVDD

Multiple logistic regression was performed, initially considering age, sex, E/e’, LVMi, lateral e’, septal e’, E/A, E/e’, E DT, diastolic and systolic blood pressure (all *p* < 0.5 to predict LVDD) (Table 5). Then using backward selection, the final model included lateral e’, LVMi and LAVi. The sensitivity and specificity and accuracy to predict LVDD was 100%. However, using leave‐one‐out cross‐validation, the accuracy was 79%, with sensitivity, and specificity of 67% and 84%.

**TABLE 5:** Multiple logistic regression to predict diastolic dysfunction.

	Univariable analysis		Multivariable analysis
Parameter	Unit OR	Significance	Significance
age	**1.08**	**0.01**	NS
Sex	2.15	0.40	NS
Diastolic BP	1.05	0.36	NS
Systolic BP	1.03	0.13	NS
E/A	0.2	0.22	NS
E DT	1.005	0.31	NS
E/e’	**1.3**	**0.05**	NS
e’ septal	**0.59**	**0.02**	NS
e’ lateral	**0.57**	**0.01**	**<0.001**
LVMi	1.02	0.42	**<0.001**
LAVi	**1.07**	**0.03**	**<0.001**

*Note*: Multivariate logistic regression to predict any LVDD by TTE, using all clinical and CMR variables with univariate *p* < 0.5, and subsequent backward selection to obtain multivariate predictors (with 100% accuracy for LVDD). Model: Ln[p/(1‐p)] = 791 + 14*LAVi–9.4*LVMi–119*Lat e’, with p the probability of LVDD.

## 4 DISCUSSION

The main findings of our study are an agreement on the presence of DD of 82% when grading LV DD by TTE and CMR, and 71% absolute agreement. Using multiple logistic regression including lateral e’, LV mass, and LA volume, we obtained a model with 100% agreement on the presence of DD, with reduced performance when using leave‐one‐out analysis. The individual metrics by CMR (E, A, E/A, e’, E/e’ even LAVi) correlated moderately to strongly with TTE (*r* > 0.6), showing similar correlations as found in prior studies (Fujikura et al., 2024; Ramos et al., 2020; Rathi et al., 2008; Seemann et al., 2018) and were even stronger for the more time‐concordant studies (*r* > 0.7).

For example, compared to Ramos et al. (2020), our correlations of TTE and CMR (<45 days apart) showed very similar strengths for A, e’, E/e’, and LAVi, while E showed a stronger correlation in our study (0.68 vs. 0.55) and E/A showed a worse correlation (0.75 vs. 0.94). However, we measured E and A to have less bias, while e’ had greater bias in our study. Their e’ method used an undersampled radial PC approach (Fyrdahl et al., 2020) with a low VENC, and high frame‐rates. Compared to the very recent study of Fujikura et al. (2024), our correlations of TTE and CMR (<45 days apart) showed worse correlations for E (*r* = 0.68 vs. *r* = 0.78), A (*r* = 0.78 vs. *r* = 0.90), and E/A (*r* = 0.75 vs. *r* = 0.82), but stronger for e’ (*r* = 0.75 vs. *r* = 0.64) and E/e’(0.8 vs. 0.54) and LAVi (*r* = 0.79 vs., *r* = 0.61). Their e’ measurement was also based on 4‐chamber cine analysis, but used semi‐automated approach that has not been published.

When using CMR criteria analogous to TTE, CMR detected more disease than TTE, which may be false positives or may mean that CMR is more sensitive than TTE. TTE has good specificity but has a reported sensitivity to elevated pressure as low as 50% (van de BovenKamp et al., 2021). e’ represents increased stiffness which is known to be age‐related (De Sutter et al., 2005). CMR‐derived e’ was more strongly associated with age than TTE‐derived e’, potentially indicating that it is more robustly measured by CMR.

The percentage accuracy of CMR in this study for exact LVDD grading (71%) can be compared to test–retest studies using TTE for LV diastolic function evaluation, e.g. which reported 84% agreement for exact diastolic grade (Bahrami et al., 2021).

Our approach uses standard MRI methods, widely available, but innovatively applied. For example, there is no data on deep learning tracking of the mitral valve annulus as a surrogate for e’, although this has been studied using feature‐tracking (Seemann et al., 2018), since most methods use tissue‐phase mapping. e’ was lower by CMR, and this may be partly related to increased temporal resolution of TTE, or differences due to the TTE beam angle. Most prior CMR studies used short‐axis basal PC to measure E and A. However, this approach risks missing peak E or A, as the basal slice moves during diastole. Our approach (long‐axis PC) might be less accurate at obtaining true peak valvular velocities, if the peak velocity is not centered within the valve. It is also possible that it might be more accurate at finding the peak E and A velocities–if the short‐axis slice chosen does not contain these velocities. A recent study (Xiang et al., 2024) compared the long‐axis and short‐axis PC approaches and found strong albeit imperfect agreements for E (*r* = 0.76, slope = 0.88) and A (*r* = 0.61, slope = 0.75) with slightly larger E and A values using long‐axis PC. TTE itself clearly is limited in its choice of the “range gate” location interrogated for E and A measurement, and uses a long‐axis view. In this aspect, the long‐axis PC approach more closely approximates TTE's method. LAVi was measured conventionally, and we found larger LA volumes by CMR, similar to previous reports (Kuhl et al., 2012).

CMR has better reproducibility and accuracy vs. TTE in general and is less operator dependent. For example, one study of interobserver variability of maximum LAVI for TTE found coefficient of variation (SD/mean value) of 12% (Olsen et al., 2022) versus 6% (Nacif et al., 2013) for CMR. Another older study defined the coefficient of variation for TTE to be approximately 7% for E/A, 10% for E (Palmieri et al., 2003) for inter‐study variability within 24 h; another study (Bahrami et al., 2021) measured E to have inter‐study coefficient of variability of 20%, while E/e’ had a coefficient of variation of 30%. These inter‐study variations of TTE (and CMR) place upper limits on the level of agreement between CMR and TTE, and may be partly physiological. Here, we measured scan‐rescan variability (within the same MRI exam) to be 9%, 7%, and 15% (E, A, and e’) by CMR.

We expect that CMR will be quite useful in LVDD. While CMR cannot replace TTE, which is lower‐cost, portable and accessible, the current trend in CMR is towards shorter exams (Raman et al., 2022), lower field‐strengths (Campbell‐Washburn et al., 2019) (Zhao et al., 2024), and lower‐cost scanners (Selvaganesan et al., 2024) for greater accessibility. All of this increases the probability of CMR's use and its important role in the pivotal diagnosis of DD. The LVDD protocol described here requires less than 5 min.

Multiple logistic regression performed better than use of CMR‐cutoffs for identifying LVDD. In this task, lateral e’ was particularly important, combined with LAVi and LVMi, as a CMR variable used for predicting the presence of LVDD with 100% accuracy (although 79% using leave‐one‐out analysis). Interestingly, a recent paper identified LAVi and LVMi as two CMR variables which could be combined to predict elevated LV filling pressure (Garg et al., 2022), while CMR‐derived e’ was not available in their study.

### 4.1 Limitations

The cohort size was small, and while this unlikely impacts the linear relationships between TTE and CMR‐derived DD parameters, a larger population would certainly show more confidently the diagnostic utility of CMR‐derived DD evaluation. The delay between the TTE and the CMR imaging (average 44 days) was a limitation especially concerning since DD is a condition that is highly dependent on volume status (which can change on the time scale of days). As exhibited in the sub‐analysis presented here, a shorter time gap between studies strengthened the TTE/CMR correlations. Also, the CMR‐derived evaluation of DD graded benefited from the information on TR jet velocities by TTE. However, this is a limitation of CMR itself–since it cannot evaluate these jet velocities. Currently, new approaches for measuring RV systolic pressure gradients (Driessen et al., 2018; Feneis et al., 2018; Lamy et al., 2024) are being developed, and this may be feasible. Improved temporal resolution might have provided higher CMR‐derived e's closer to TTE, since in another study, e’ increased by 14%, when the temporal resolution was decreased from 30 to 16 ms, while E and A were unchanged (Xiang et al., 2024). Finally, in this CMR study, we excluded patients with data corrupted by arrhythmias, although this is also a challenge for TTE (Nagueh et al., 2016).

In conclusion, CMR is a sensitive method for diastolic function evaluation, providing overall strong agreement to TTE metrics.

## AUTHOR CONTRIBUTIONS

**Jérôme Lamy:** Conceived and designed research, performed experiments, analyzed data, interpreted results of experiments, prepared figures, drafted manuscript, edited and revised manuscript, approved final version of manuscript. **Jie Xiang:** Performed experiments, analyzed data, interpreted results of experiments, prepared figures, drafted manuscript, edited and revised manuscript, approved final version of manuscript. **Nimish Shah:** Performed experiments, analyzed data, interpreted results of experiments, prepared figures, drafted manuscript, edited and revised manuscript, approved final version of manuscript. **Jennifer M. Kwan:** Performed experiments, analyzed data, interpreted results of experiments, prepared figures, drafted manuscript, edited and revised manuscript, approved final version of manuscript. **Yekaterina Kim:** Performed experiments, analyzed data, interpreted results of experiments, prepared figures, drafted manuscript, edited and revised manuscript, approved final version of manuscript. **Krishna Upadhyaya:** Performed experiments, analyzed data, interpreted results of experiments, prepared figures, drafted manuscript, edited and revised manuscript, approved final version of manuscript. **Samuel W. Reinhardt:** Analyzed data, interpreted results of experiments, prepared figures, drafted manuscript, edited and revised manuscript, approved final version of manuscript. **Judith Meadows:** Conceived and designed research, performed experiments, analyzed data, interpreted results of experiments, prepared figures, drafted manuscript, edited and revised manuscript, approved final version of manuscript. **Robert L. McNamara:** Interpreted results of experiments, prepared figures, drafted manuscript, edited and revised manuscript, approved final version of manuscript. **Lauren A. Baldassarre:** Conceived and designed research, performed experiments, analyzed data, interpreted results of experiments, prepared figures, drafted manuscript, edited and revised manuscript, approved final version of manuscript. **Dana C. Peters:** Conceived and designed research, performed experiments, analyzed data, interpreted results of experiments, prepared figures, drafted manuscript, edited and revised manuscript, approved final version of manuscript.

## FUNDING INFORMATION

The authors acknowledge funding from NIH: NIH 1R01HL144706, Development of MR‐derived parameters of LV diastolic function: Validation and Comparison to LV and LA fibrosis.

## CONFLICT OF INTEREST STATEMENT

In relation to the current data, the authors declare that no conflicts of interest exist.

## ETHICS STATEMENT

The study was approved by our local IRB, the Yale Human Investigation Committee, and all subjects provided written and informed consent.
